# Supplementary figures and images for: Ubiquitin is directly linked via an ester to protein-conjugated mono-ADP-ribose
Source: EMBO J. 2025 Feb 25;44(8):2211–31. doi: 10.1038/s44318-025-00391-7 (PMC12000418; doi:10.1038/s44318-025-00391-7)

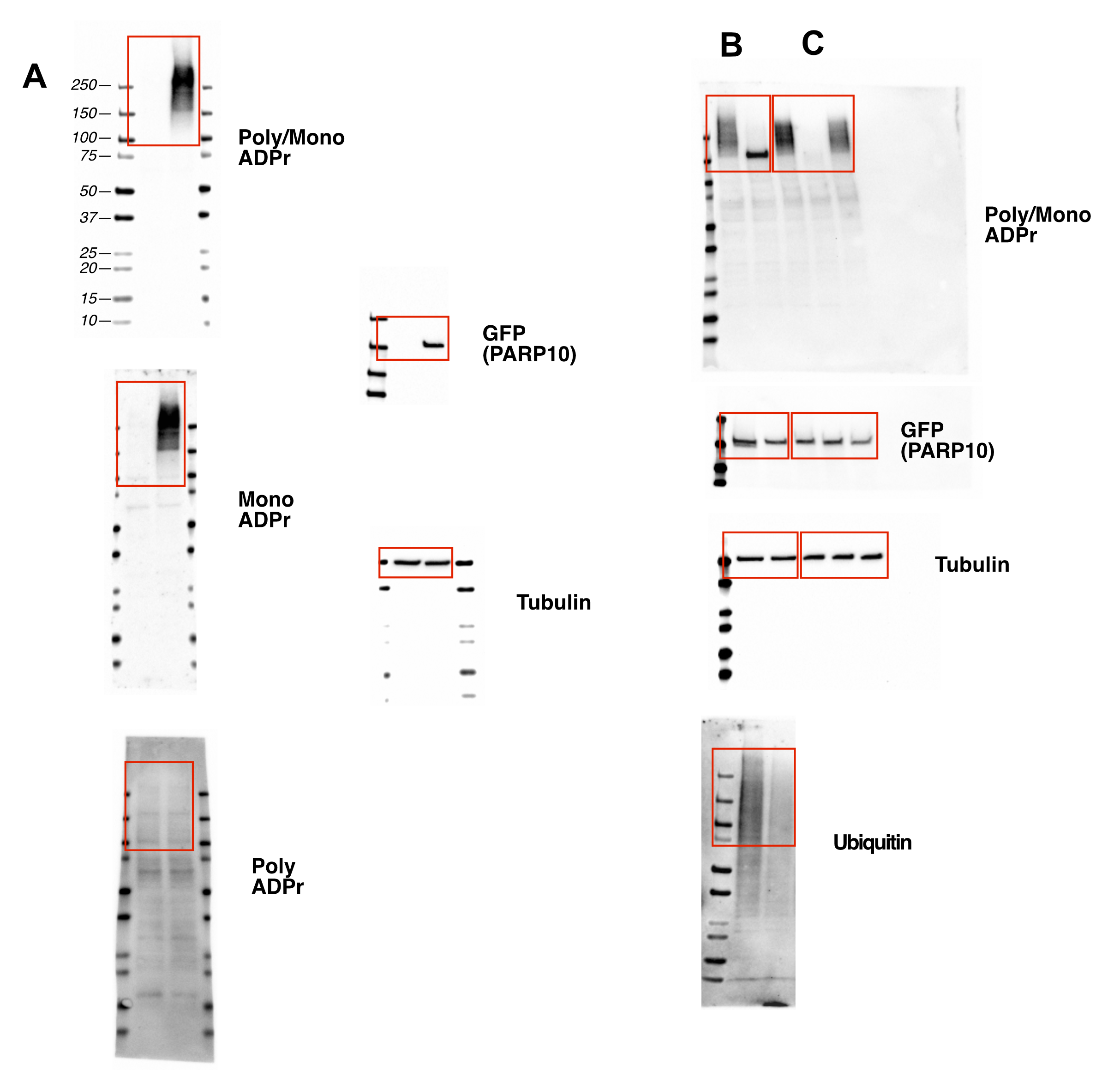

Supplement: Supplementary file 2 — Source data Fig. 1 [file 44318_2025_391_MOESM2_ESM.zip › SD Figure 1/Fig1ABC.png]

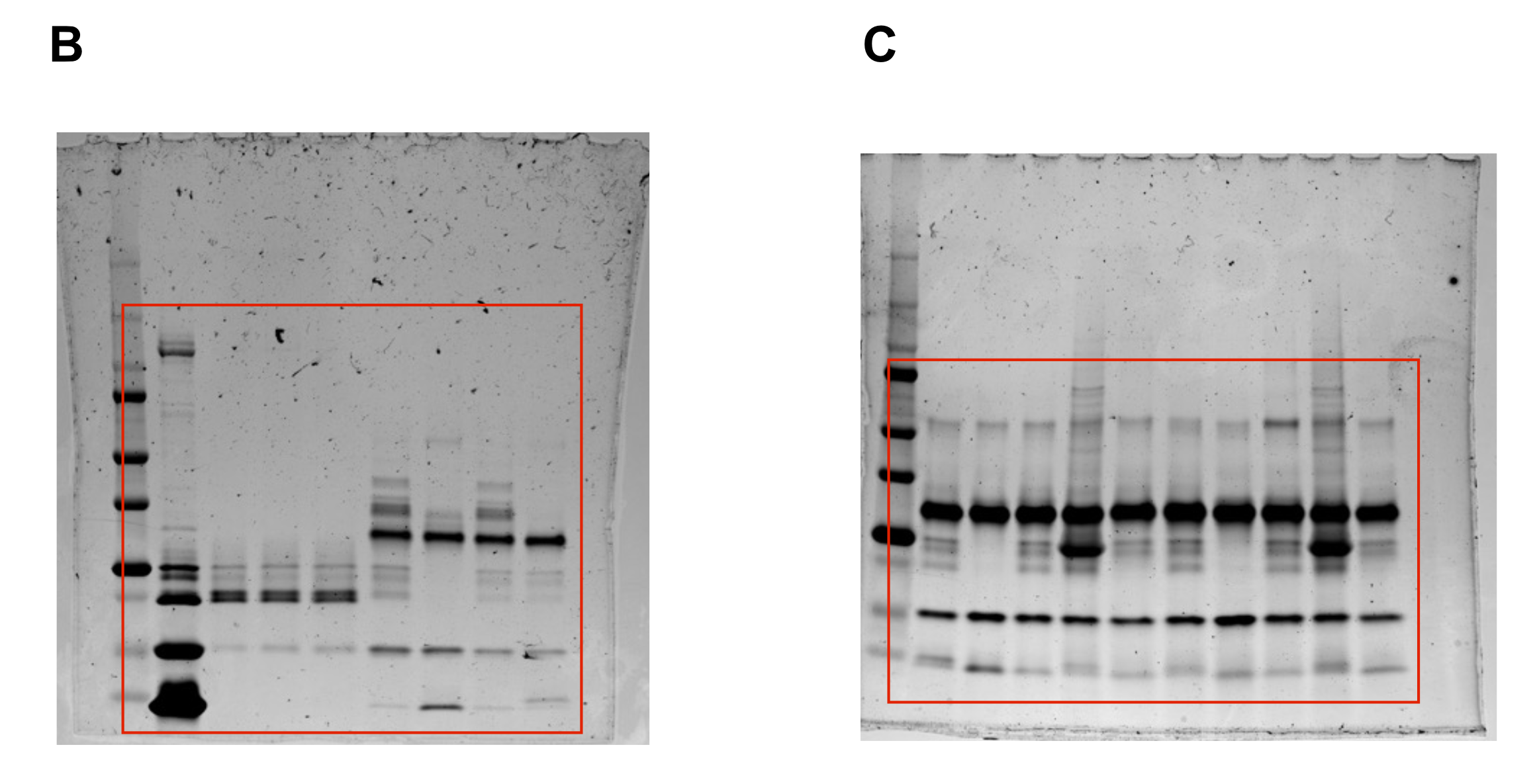

Supplement: Supplementary file 3 — Source data Fig. 2 [file 44318_2025_391_MOESM3_ESM.zip › SD Figure 2/Fig2BC.png]

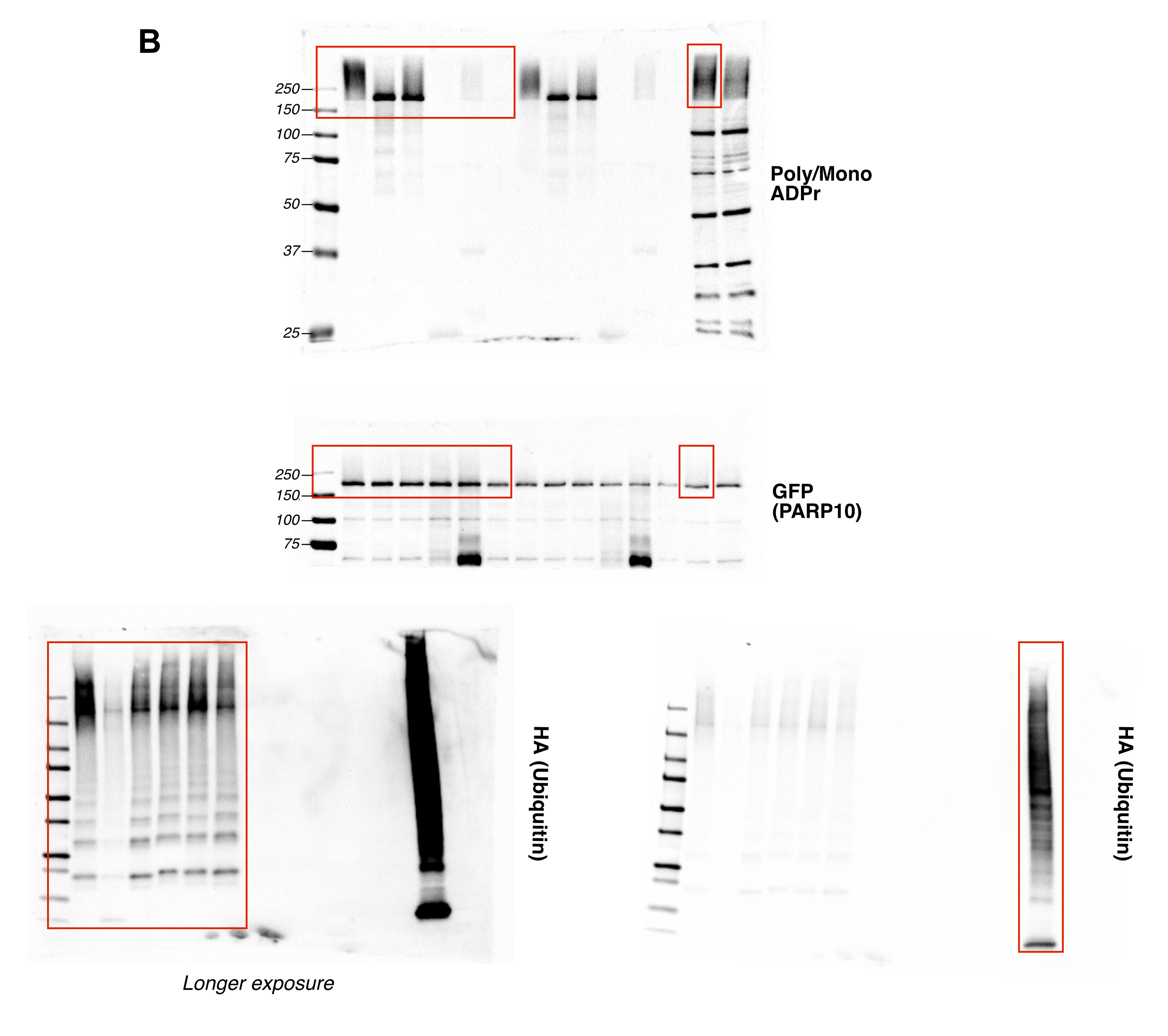

Supplement: Supplementary file 4 — Source data Fig. 3 [file 44318_2025_391_MOESM4_ESM.zip › SD Figure 3/Fig3B.png]

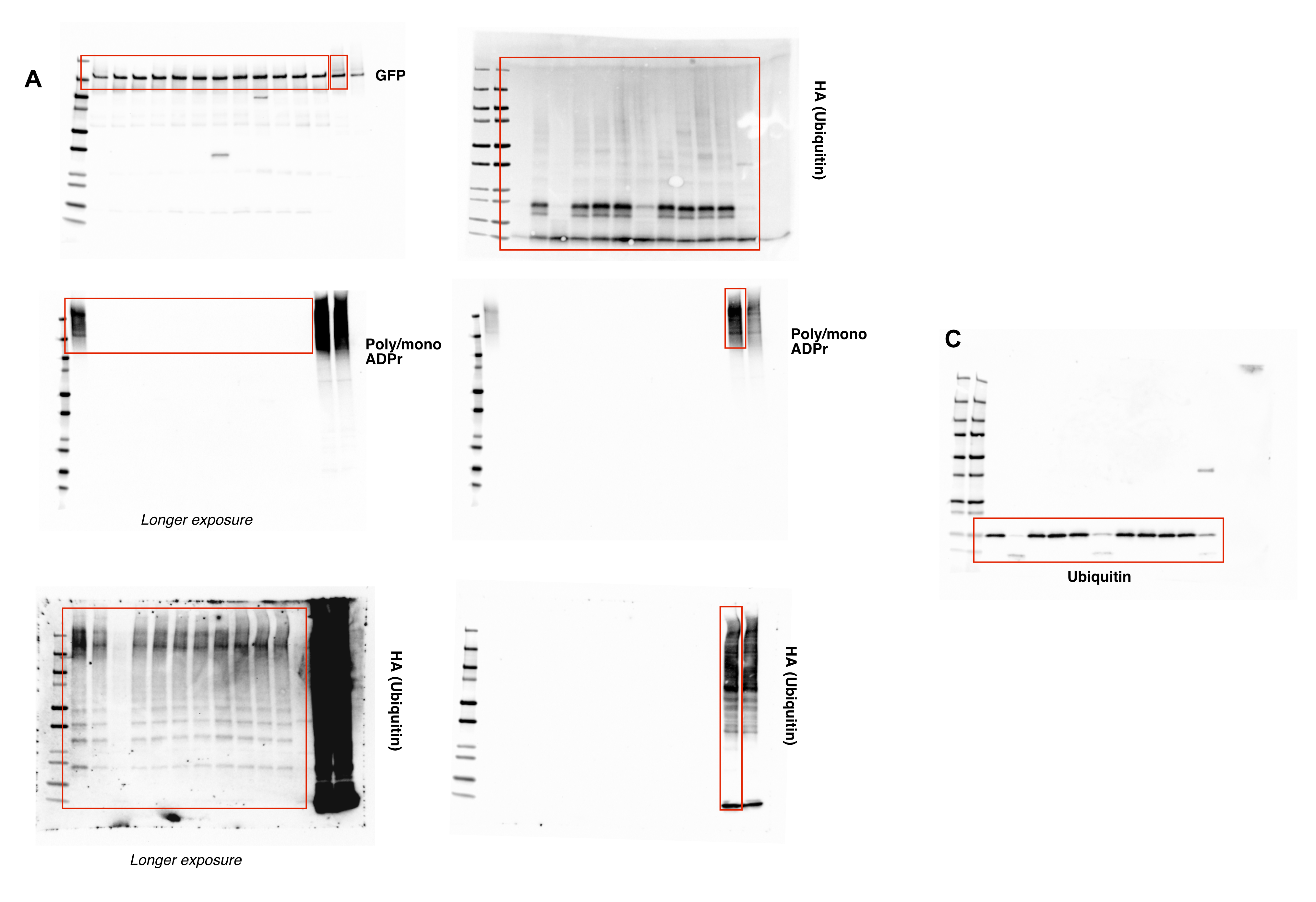

Supplement: Supplementary file 5 — Source data Fig. 4 [file 44318_2025_391_MOESM5_ESM.zip › SD Figure 4/Fig4AC.png]

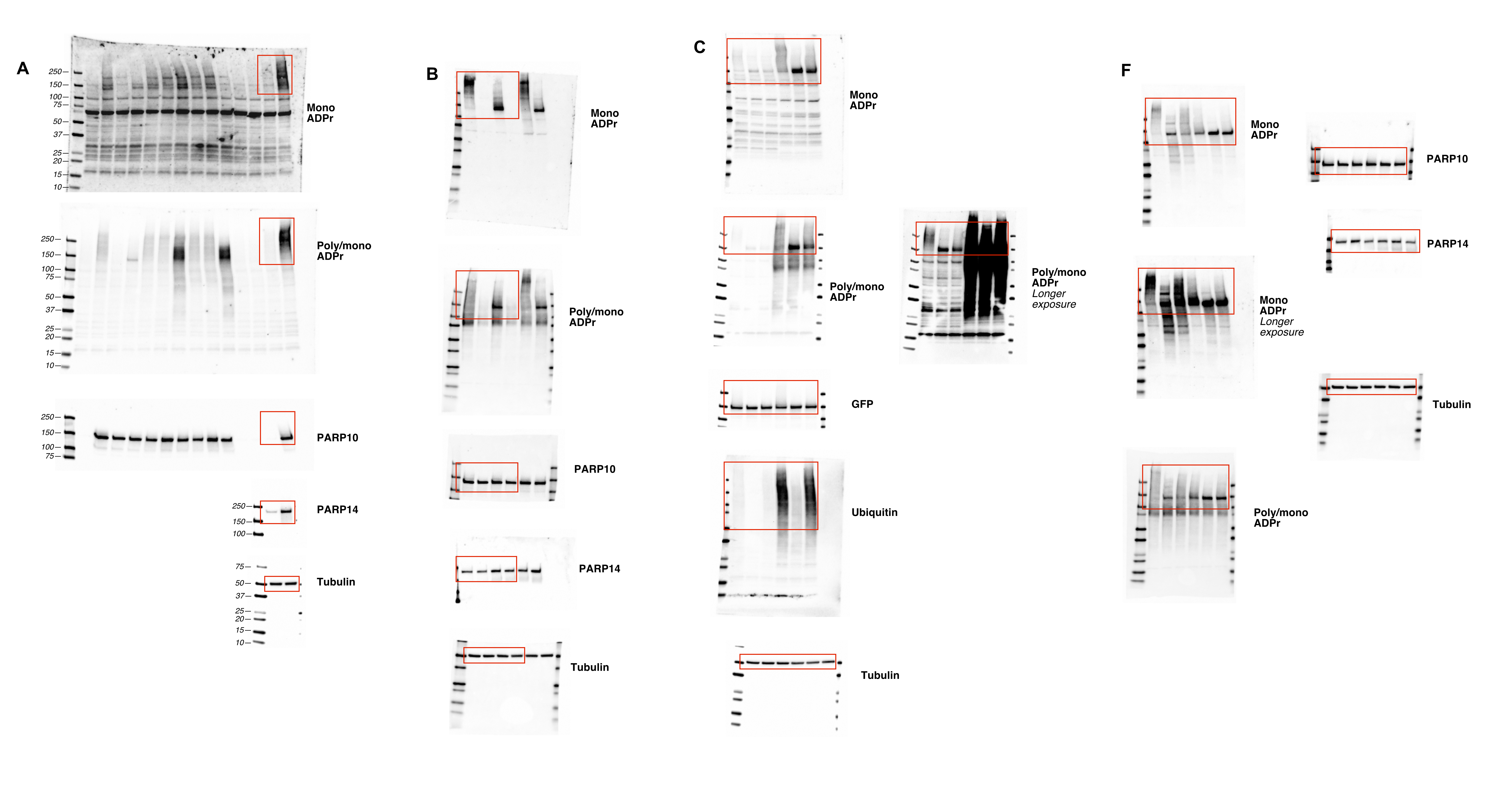

Supplement: Supplementary file 6 — Source data Fig. 5 [file 44318_2025_391_MOESM6_ESM.zip › SD Figure 5/Fig5ABCF.png]

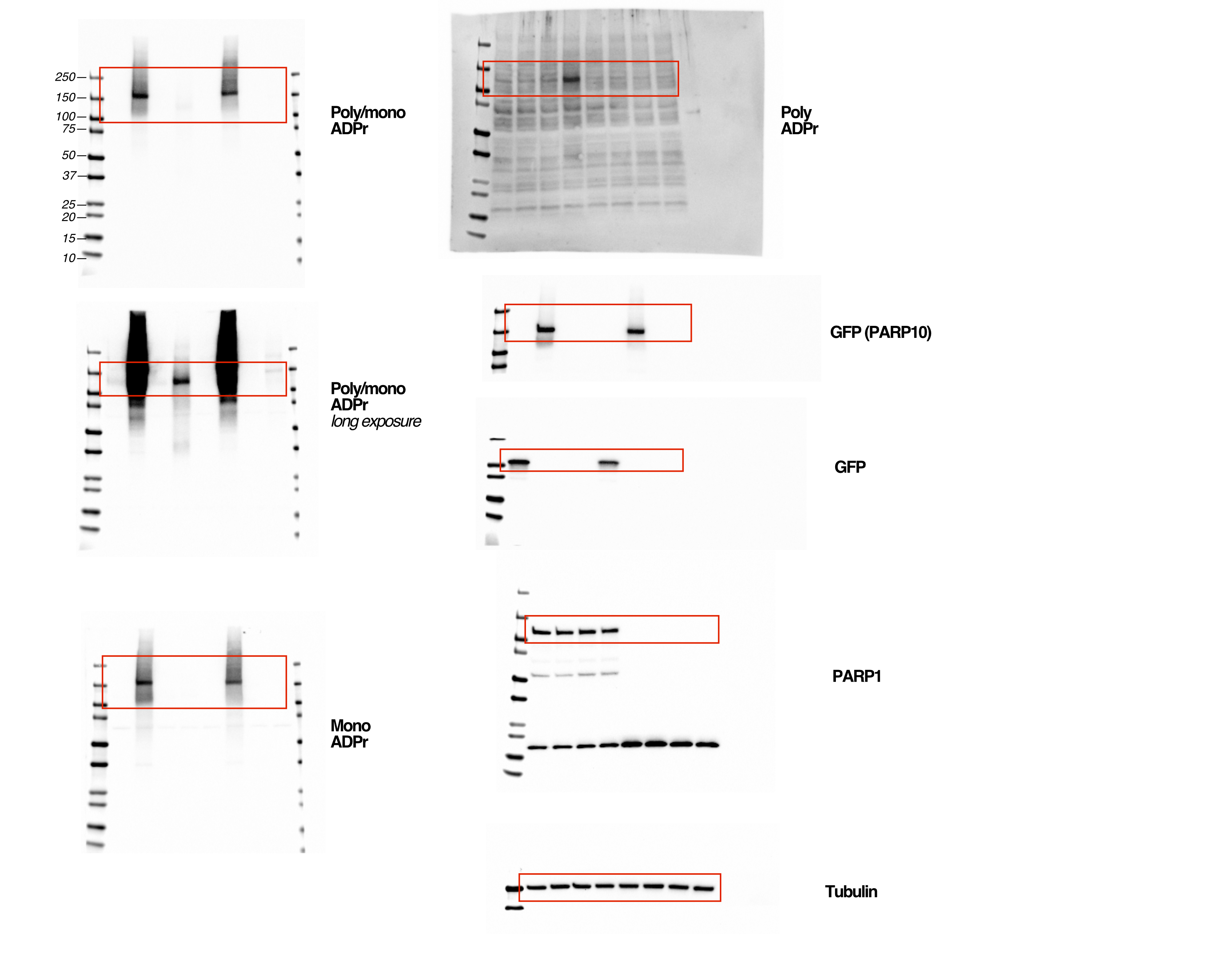

Supplement: Supplementary file 7 — Figure EV Source Data [file 44318_2025_391_MOESM7_ESM.zip › EV_Source Data/EV1.png]

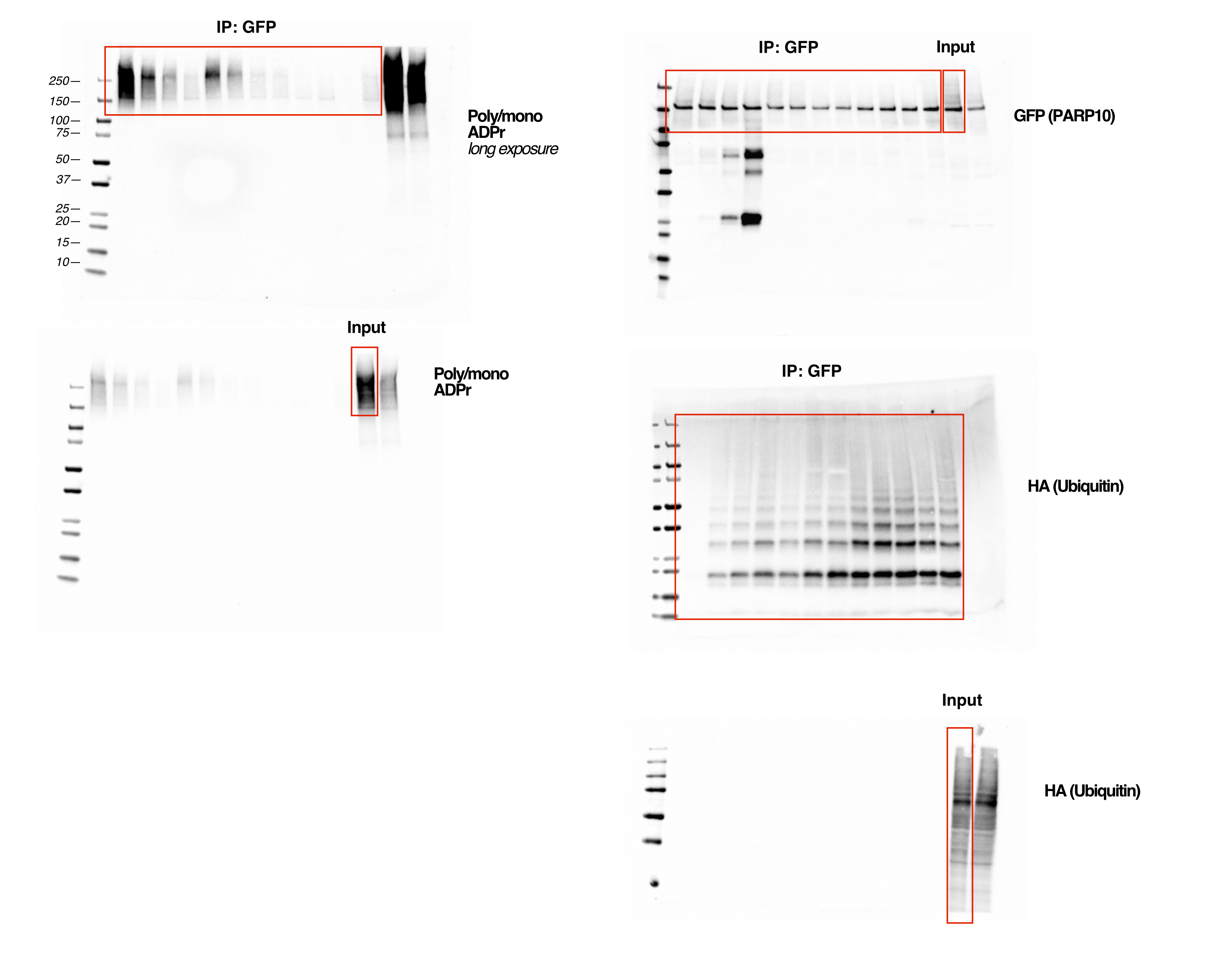

Supplement: Supplementary file 7 — Figure EV Source Data [file 44318_2025_391_MOESM7_ESM.zip › EV_Source Data/EV3.png]

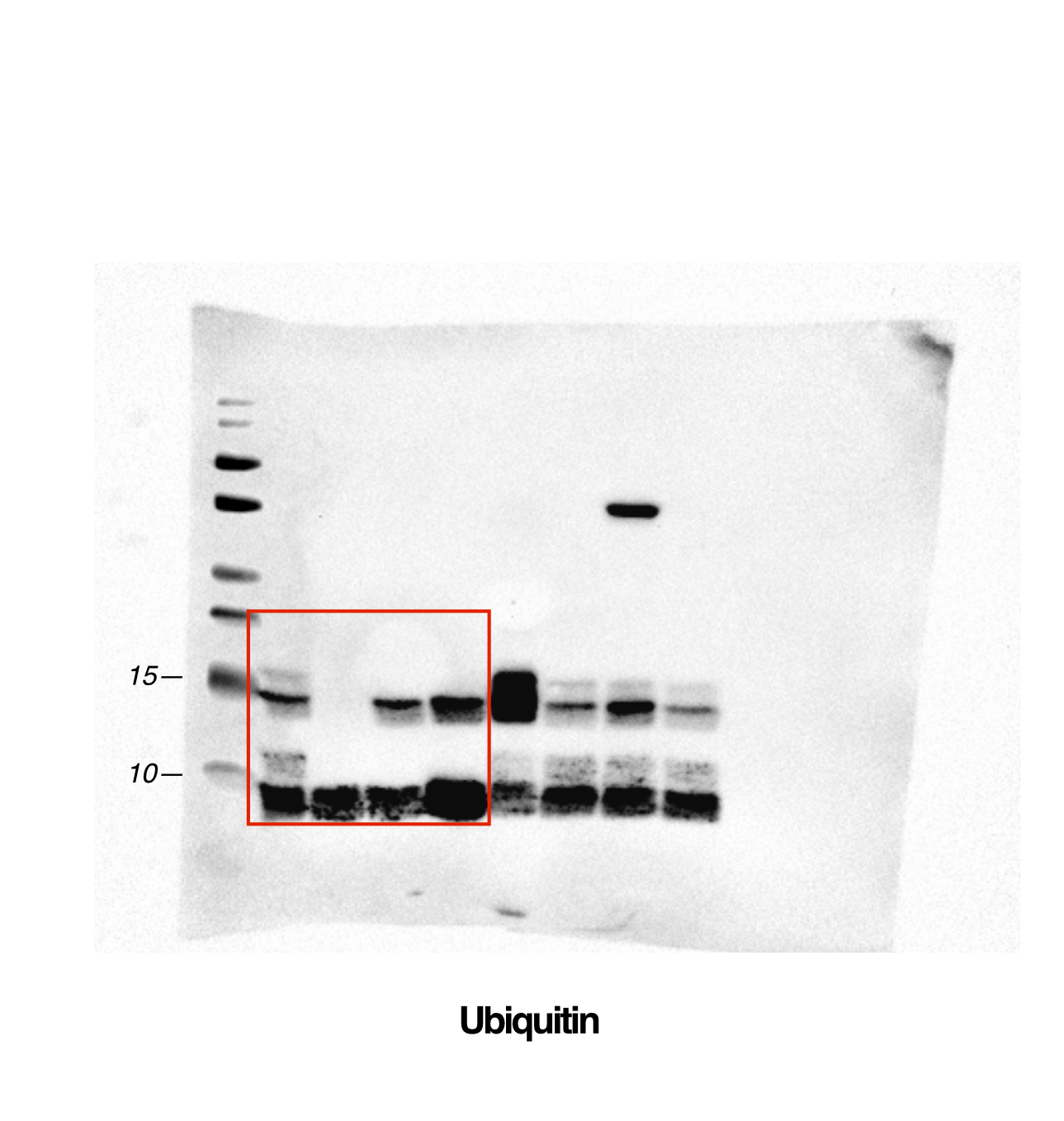

Supplement: Supplementary file 7 — Figure EV Source Data [file 44318_2025_391_MOESM7_ESM.zip › EV_Source Data/EV2_B.png]

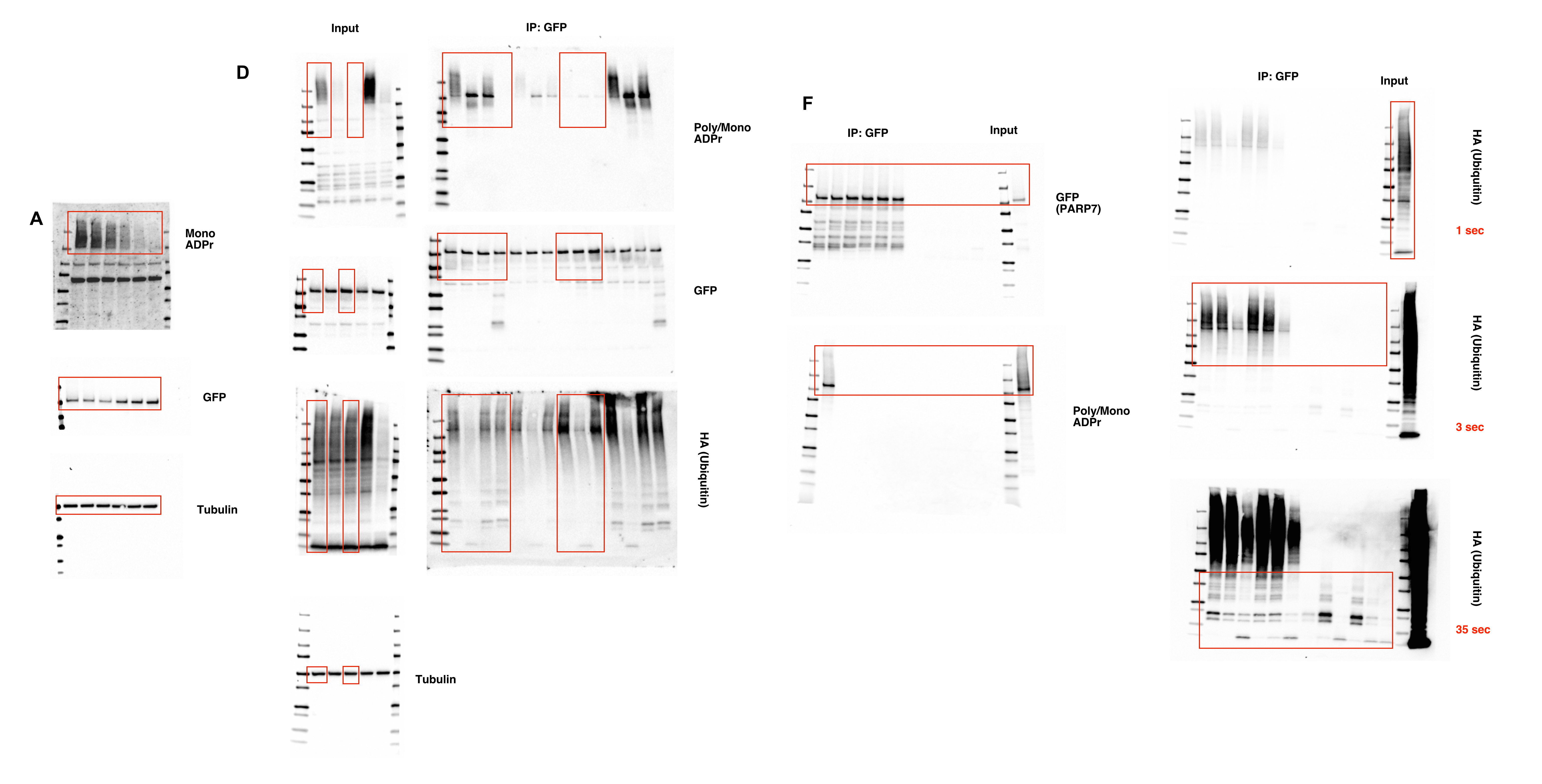

Supplement: Supplementary file 7 — Figure EV Source Data [file 44318_2025_391_MOESM7_ESM.zip › EV_Source Data/EV4_ADF.png]

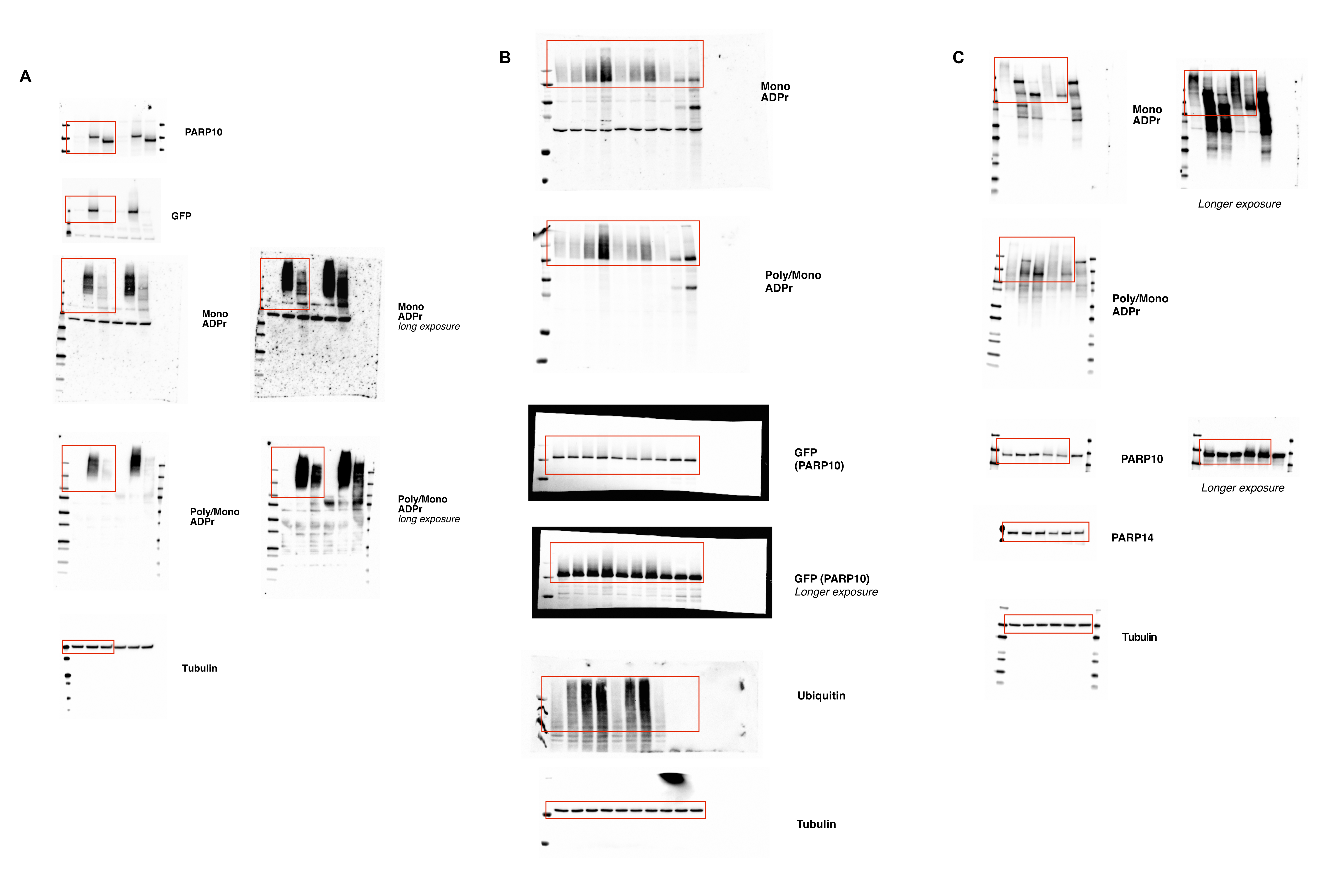

Supplement: Supplementary file 7 — Figure EV Source Data [file 44318_2025_391_MOESM7_ESM.zip › EV_Source Data/EV5_ABC.png]
