# Supplementary material for: Ubiquitin is directly linked via an ester to protein-conjugated mono-ADP-ribose
Source: EMBO J. 2025 Feb 25;44(8):2211–31. doi: 10.1038/s44318-025-00391-7 (PMC12000418; doi:10.1038/s44318-025-00391-7)
Supplement: Supplementary file 8 — Expanded View Figures [file 44318_2025_391_MOESM8_ESM.pdf]

Expanded View Figures

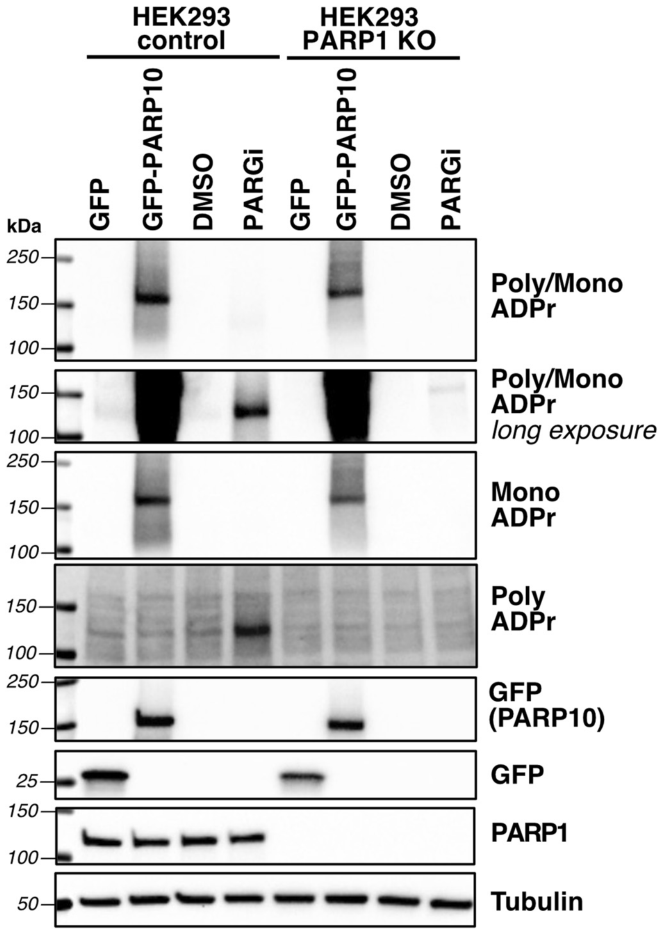

Figure EV1. Evaluating specificity of poly-ADPr, mono-ADPr, and poly/mono-ADPr antibodies.

HEK 293 control and PARP1 KO cells were transiently transfected with GFP or GFP-PARP10 for 24 h, or treated with PARG inhibitor (PDD00017273, 1 μM) for 30 min, followed by western blotting and probing for poly/mono ADPr (Cell Signaling Technology: E6F6A), mono-ADPr (Bio-Rad: HCA354), or poly-ADPr (Millipore Sigma: MABE1031). Representative western blot from $n = 2$ biological replicates. Source data are available online for this figure.

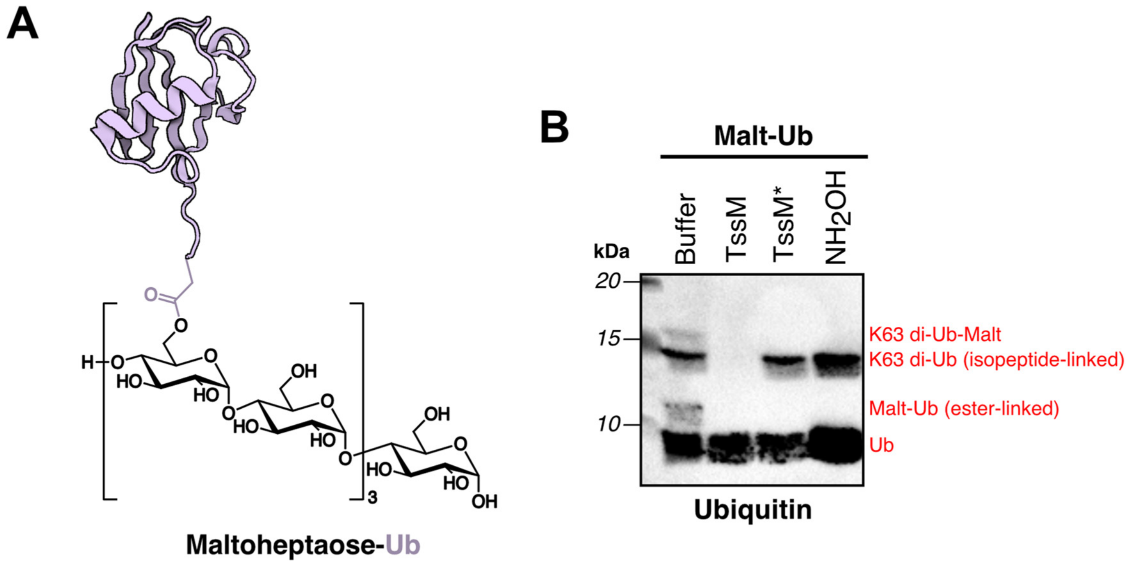

Figure EV2. TssM* is highly specific for removal of ester-linked maltoheptaose-Ub.

(**A**) Structure of maltoheptaose-Ub (Malt-Ub) with an ester-linked Ub on the C6 hydroxyl group of glucose. (**B**) Malt-Ub was incubated with 2 μM TssM, 2 μM TssM*, or 1 M NH_2OH (pH 7.5) for 1 h at 37 °C. Representative western blot from $n = 3$ biological replicates. Source data are available online for this figure.

 © The Author(s)

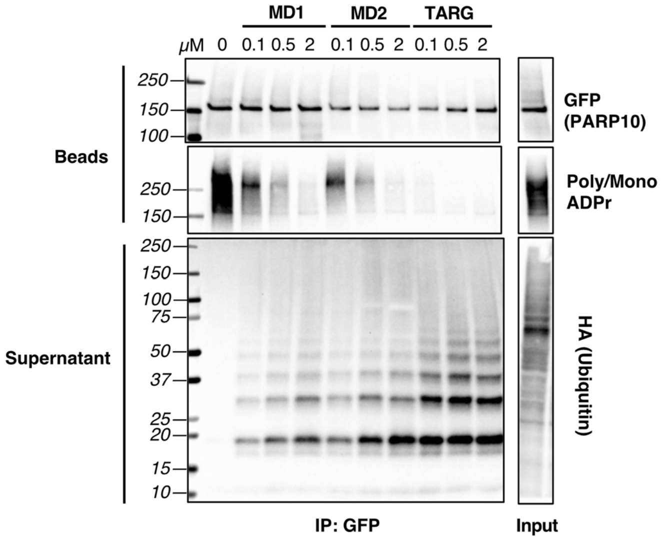

Figure EV3. Screening macrodomain-based ADPr hydrolases against PARP10 MARylation.

GFP-PARP10, from doxycycline-induced HEK 293 cells transfected with HA-Ub, was immunoprecipitated with GFP-trap beads, washed stringently (7 M Urea, 1% SDS), and beads were treated with a dose-response of MacroD1 (MD1), MacroD2 (MD2) or terminal ADP-Ribose protein glycohydrolase (TARG). The supernatant and bead fractions were separated and subjected to western blotting. Representative western blot from $n = 2$ biological replicates. Source data are available online for this figure.

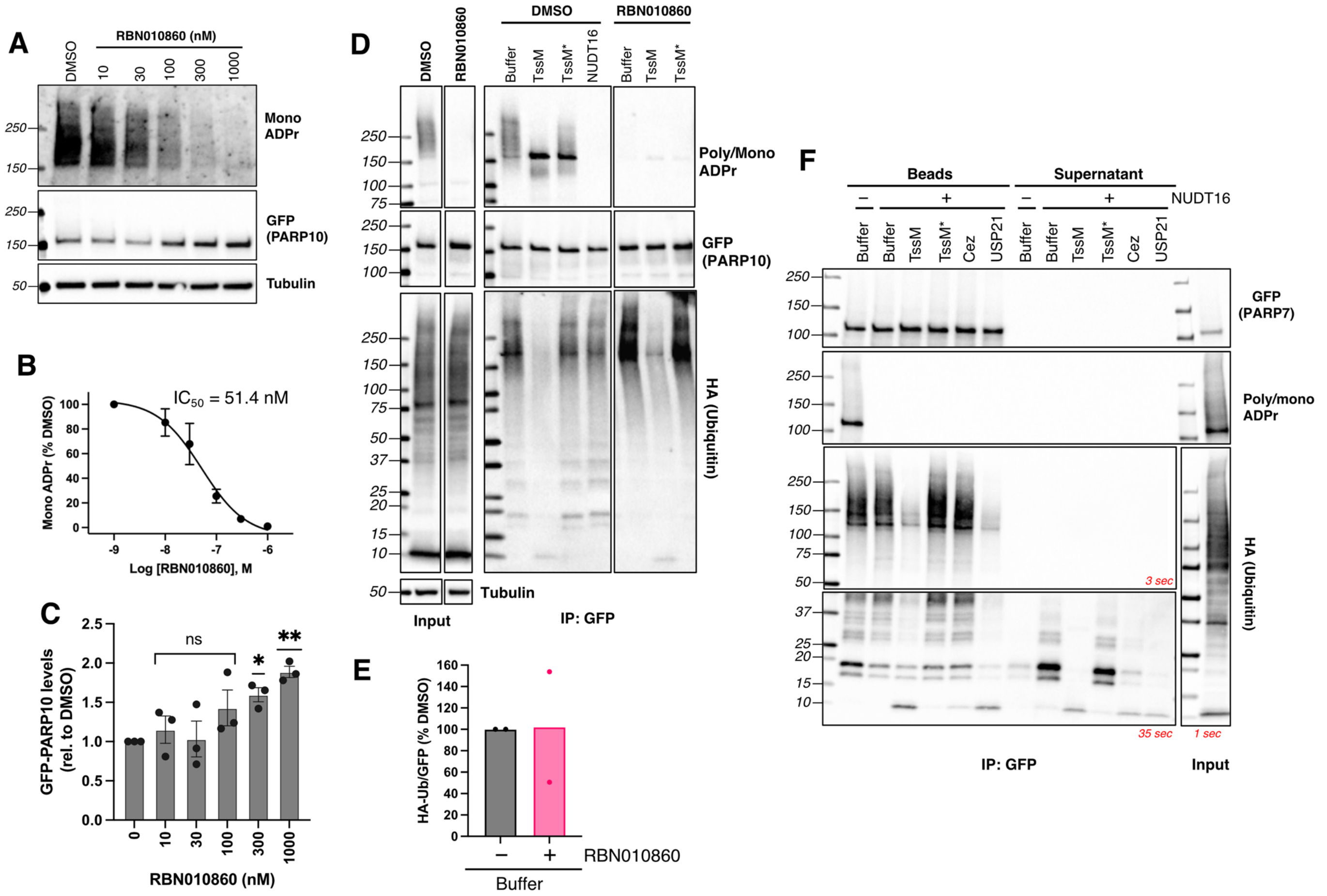

Figure EV4. PARP10 contains isopeptide-linked polyUb that is not dependent on MARylation, and PARP7 contains K11-linked MARUbylation.

(**A**) HEK 293 cells with doxycycline-inducible GFP-PARP10 were co-treated with doxycycline (10 µg/ml) and a dose-response of RBN010860 for 24 h, followed by western blotting. Quantification of the IC_{50} value of RBN010860 (**B**) and GFP-PARP10 levels (**C**) from (**A**); *n* = 3 biological replicates. Asterisks indicate statistical significance (ns: not significant). For (**C**), 300 nM RBN010860: **P* = 0.0219, 1000 nM RBN010860: ***P* = 0.0064, calculated using a one-sample t and Wilcoxon test relative to vehicle treatment. (**D**) GFP-PARP10 dox-inducible HEK 293 cells were transfected with HA-Ub and treated with DMSO or 1 µM RBN010860 for 24 h, followed by immunoprecipitation with GFP-trap beads, stringent washing (7 M Urea, 1% SDS), and on-bead treatment with TssM (1 µM) or NUDT16 (10 µM). (**E**) Quantification of the HA-Ub signal (normalized to GFP) from buffer-treated samples from DMSO or RBN010860 treatment; *n* = 2 biological replicates. (**F**) GFP-PARP7 and HA-Ub were transiently co-expressed in HEK 293 T cells and immunoprecipitated with GFP-trap beads, followed by stringent washing (7 M Urea, 1% SDS), and treatment with NUDT16. The beads and supernatant were separated and further treated with 2 µM TssM, 2 µM TssM*, 0.2 µM Cezanne, or 1 µM USP21, followed by western blotting (representative image of *n* = 1 biological replicate). Source data are available online for this figure.

 © The Author(s)

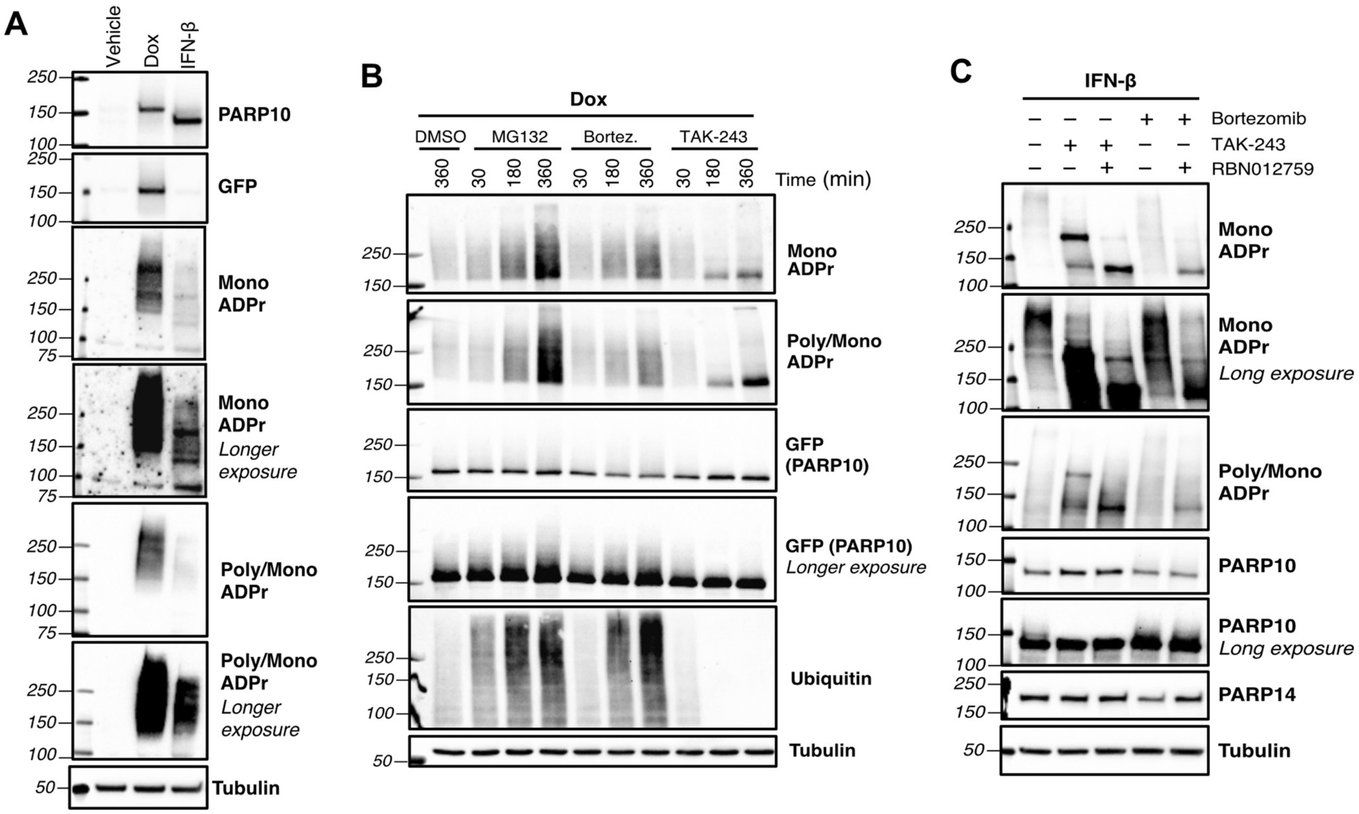

Figure EV5. Inhibition of the ubiquitin-proteasome pathway alters dox-inducible GFP-PARP10 and IFN-β-inducible MARUbylation.

(**A**) HEK 293 GFP-PARP10 doxycycline-inducible cells were treated with doxycycline (10 μg/ml) or IFN-β (140 U/ml) for 24 h, followed by western blotting ($n = 1$ biological replicate). (**B**) HEK 293 GFP-PARP10 doxycycline-inducible cells were treated with doxycycline (10 μg/ml) for 24 h, followed by a time course with MG132 (10 μM), bortezomib (0.1 μM), and TAK-243 (1 μM). Cells were lysed in CLB followed by western blotting. Representative western blot from $n = 3$ biological replicates. (**C**) PARP1 KO HEK 293 cells were treated with IFN-β (140 U/ml) and RBN012759 (0.1 μM) for 24 h, followed by 3-h treatment with MG132 (10 μM) or bortezomib (0.1 μM). Cells were lysed in CLB with 1% SDS followed by western blotting (no boiling of samples). Representative western blot from $n = 2$ biological replicates. Source data are available online for this figure.

© The Author(s)
